# Supplementary material for: Visualizing the interfacial-layer-based epitaxial growth process toward organic core-shell architectures
Source: Nat Commun. 2024 Feb 7;15:1130. doi: 10.1038/s41467-024-45262-7 (PMC10850097; doi:10.1038/s41467-024-45262-7)
Supplement: Supplementary file 1 — Supplementary Information [file 41467_2024_45262_MOESM1_ESM.pdf]

Surporting Information

Visualizing the interfacial-layer-based epitaxial growth process toward organic core-shell architectures

Ming-Peng Zhuo[1,2,3], Xiao Wei[2], Yuan-Yuan Li[3], Ying-Li Shi[1], Guang-Peng He[1], Huixue Su[2], Ke-Qin Zhang[3], Jin-Ping Guan[3], Xue-Dong Wang[1], Yuchen Wu[2]*, Liang-Sheng Liao[1,4]**

[1]Institute of Functional Nano & Soft Materials (FUNSOM), Jiangsu Key Laboratory for Carbon-Based Functional Materials & Devices, Soochow University, 199 Ren'ai Road, Suzhou, Jiangsu 215123, P. R. China.

[2]Technical Institute of Physics and Chemistry Chinese Academy of Sciences, Beijing 100190, China.

[3]China National Textile and Apparel Council Key Laboratory for Silk Functional Materials and Technology, National Engineering Laboratory for Modern Silk, College of Textile and Clothing Engineering, Soochow University, Suzhou, Jiangsu 215123, China.

[4]Macao Institute of Materials Science and Engineering, Macau University of Science and Technology, Taipa 999078, Macau SAR, China.

These authors contributed equally: Ming-Peng Zhuo, Xiao Wei.

*E-mail: wangxuedong@suda.edu.cn (W. -D. Wang), wuyuchen@iccas.ac.cn (Y. Wu) and lsliao@suda.edu.cn (L.-S. Liao).

Supplementary Methods

1. Materials

Benzo[ghi]perylene (BGP, CAS: 191-24-2, 99%), 1,2,4,5-tetracyanobenzene (TCNB, CAS: 712-74-3, 98%), and tetrafluoroterephthalonitrile (TFP, CAS: 1835-49-0, 97%) were purchased from Sigma-Aldrich Co. The dichloromethane (DCM, analysis grade), methanol (analysis grade), *n*-hexane (analysis grade), cyclohexane (analysis grade) and ethanol (analysis grade) solvents were purchased from Sinopharm Group, China. In addition, all compounds and solvents were used without further treatment. The polytetrafluoroethylene filters (PTFE, Puradisc 25 TF, 0.1 μm) were bought from Whatman International Ltd.

2. Characterisations

The morphology and size of the organic micro/nanostructures were examined by emission scanning electron microscopy (FESEM, Carl Zeiss, Supra 55, Germany) with a 20 X-MaxN Energy Dispersive Spectrometer (EDS, Oxford Instruments, United Kingdom) dropping on an indium tin oxide (ITO) coated glass. TEM images were obtained by a transmission electron microscopy (TEM, FEI company, Tecnai G2 F20, United States). A drop of the solution was dropped on a carbon-coated copper grid, and evaporated. TEM measurement was performed at room temperature at an accelerating voltage of 100 kV. The X-ray diffraction (XRD) patterns were measured by a D/max 2400 X-ray diffractometer with Cu $K\alpha$ radiation (λ = 1.54050 Å) operated in the 2θ range from 5° to 30°, by using the samples on the quartz. Fluorescence images were recorded using a fluorescence optical microscope (Leica, DM4000M, Germany) with a spot-enhanced charge couple device (Diagnostic Instrument, Inc.). The excitation source is a mercury lamp equipped with a band-pass filter (330-380 nm for UV-light and 500~550 nm for green-light). The samples were prepared by placing a drop of solution onto a cleaned quartz, and then evaporated at room temperature.

Micro-area photoluminescence (μ-PL) spectra were collected on a homemade optical microscopy. To measure the PL spectra of individual microplate, the micro/nanostructure was excited locally with a 375 nm laser focused down to the diffraction limit. The excitation laser was filtered with a 375 nm notch filter. The light was subsequently coupled to a grating spectrometer (Princeton Instrument, ARC-SP-2356) and recorded by a thermal-electrically cooled CCD (Princeton Instruments, PIX-256E). PL microscopy images were taken with an inverted microscope (Olympus, BX43).

Supplementary Discussion

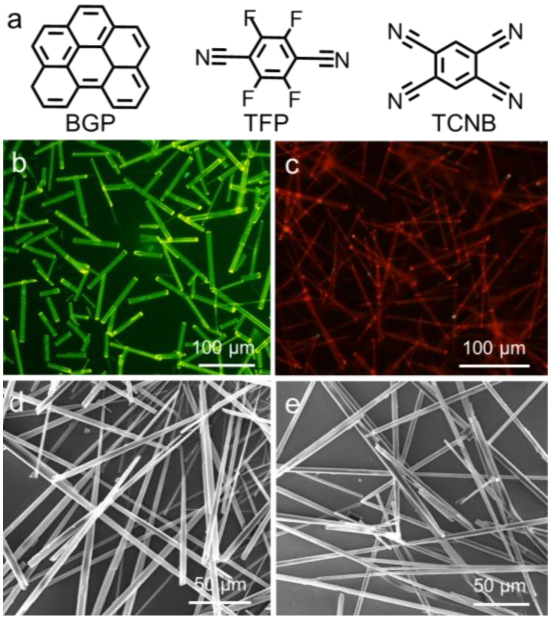

Supplementary Figure 1. (a) The molecular structure of BGP, TFP, and TCNB. FM images of (b) BTP and (c) BTB microwires with the scale bar of 100 μm. SEM images of (d) BTP and (e) BTB microwires with the scale bar of 50 μm.

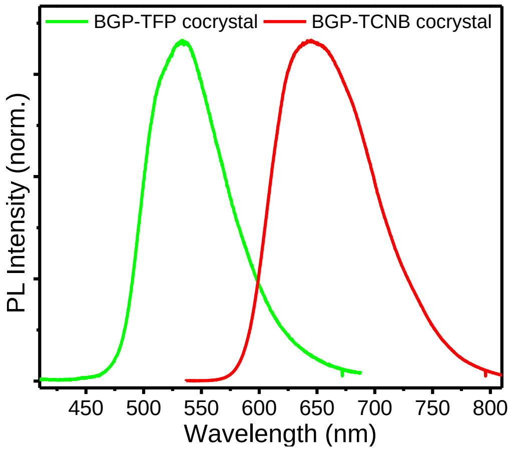

Supplementary Figure 2. The PL spectra of BTP (green line) and BTB (red line) microwires.

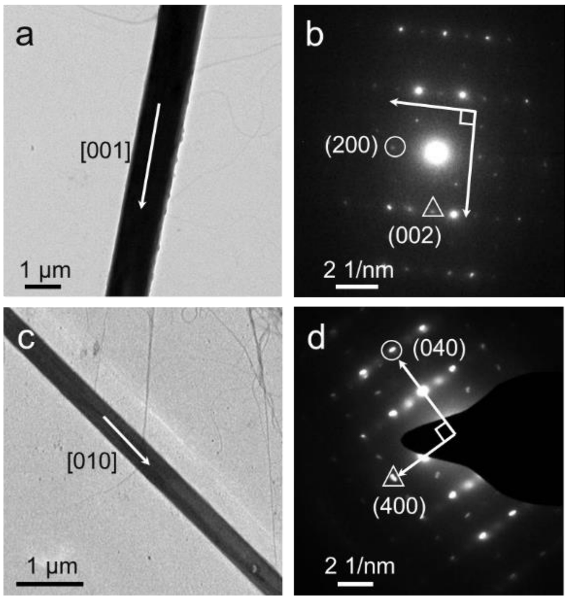

Supplementary Figure 3. (a) TEM image and (b) SAED pattern of BTP microwires. (c) TEM image and (d) SAED pattern of BTB microwires. The scale bar of the TEM images and the corresponding SAED patterns are 1 μm and 2 1/nm, respectively.

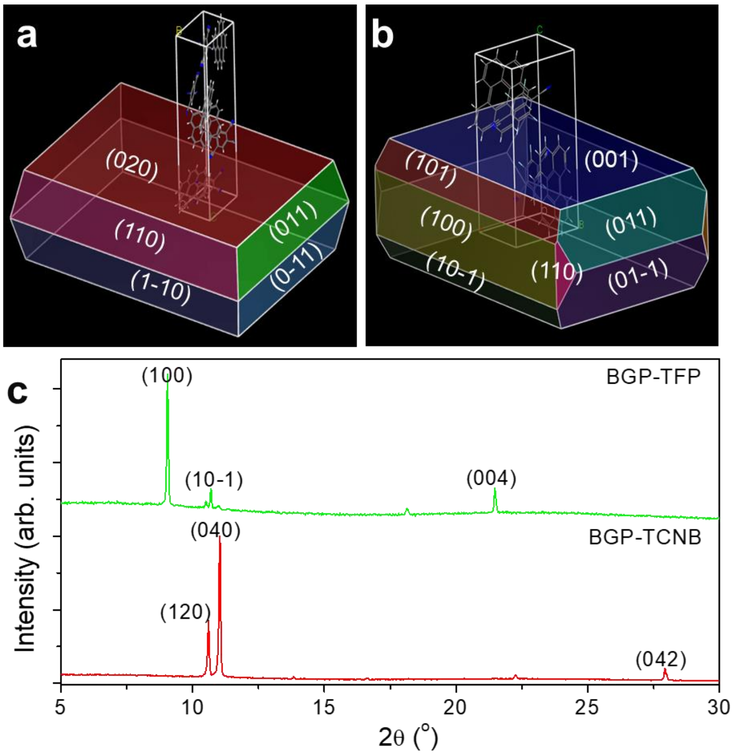

Supplementary Figure 4. Predicted growth morphology of (a) BTB and (b) BTP cocrystals. (c) The XRD patterns of BTP (green line) and BTB (red line) cocrystals.

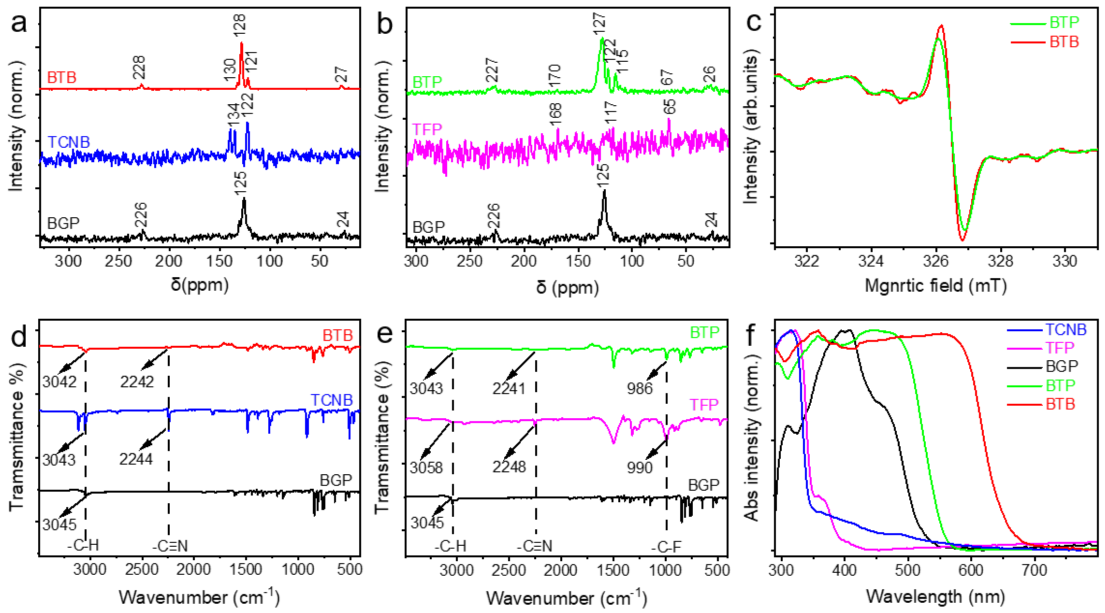

Supplementary Figure 5. (a) and (b) Solid-state ^{13}C NMR spectra, (c) ESR spectra, (d and e) IR spectra, and (e) diffuse reflection absorption spectra of these of organic BTP and BTB cocrystals.

Solid-state ^{13}C NMR results were reveals the chemical environment of the C atoms.[1] According to solid-state ^{13}C NMR results (Supplementary Figure 5a and 5b), after cocrystallization of BTB or BTP cocrystal, the chemical shift of BGP shows an obvious upfield shift from 125 to 127 or 128 ppm, indicating the increase of electron density.[2] Meanwhile, the chemical shifts of TCNB at around 134 ppm and TFP at around 117 ppm exhibit downfield shifts. These observations demonstrate the π-charge-transfer from TCNB or TFP to BGP molecules, implying the existence of intermolecular CT interactions.[3] The electron spin resonance (ESR) spectrum of BTP exhibits a strong resonance signal with *g* factor of 2.0037 (Supplementary Figure 5c). This result suggests the existence of unpaired electrons in BTP, which arises from the CT process.[4] Furthermore, the stronger ESR signal of BTB compared to that of BTP suggests a stronger CT interaction between BGP and TCNB than that between BGP and TFP. As depicted in Supplementary Figure 5d and 5e, the characteristic peak of C≡N stretching vibrations at 2244 cm^{-1} in TCNB and 2248 cm^{-1} in TFP is blue-shifted to 2242 cm^{-1} in BTB cocrystals and 2241 cm^{-1} in BTP cocrystals, respectively, suggesting a π-charge-transfer from BGP to TCNB or TFP.[5] Compared to the constituent molecules, the BTP and BTB cocrystals exhibits a broader red-shifted peak at 500 and 620 nm as verified in Supplementary Figure 5f, which is corresponding to the π-charge-transfer from BGP to TFP or TCNB.[6] As a short conclusion, the π-charge-transfer from BGP to TCNB or TFP in these prepared BTP and BTB cocrystals was verified by Fourier Transform Infrared (FT-IR) spectra, Solid-state ^{13}C NMR results, electron spin resonance (ESR) spectra, and diffuse reflection absorption spectra in Supplementary Figure 5.

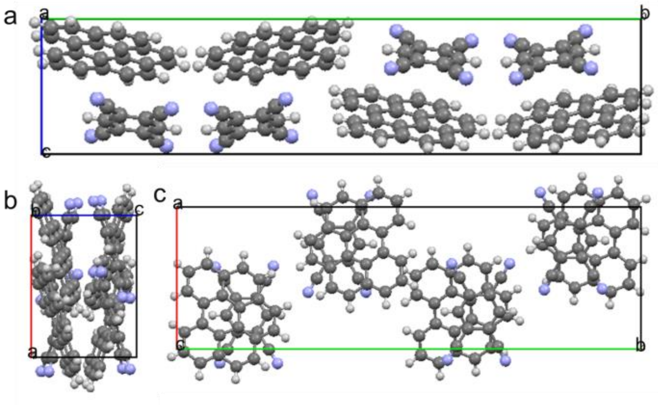

Supplementary Figure 6. Unit cell structure of BTP crystals. Molecular packing arrangement in (a) (100), (b) (010), and (c) (001) crystal planes.

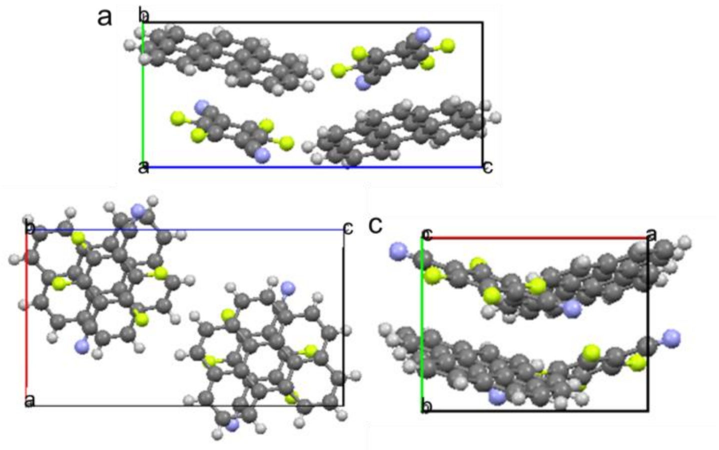

Supplementary Figure 7. Unit cell structure of BTB crystals. Molecular packing arrangement in (a) (100), (b) (010), and (c) (001) crystal planes.

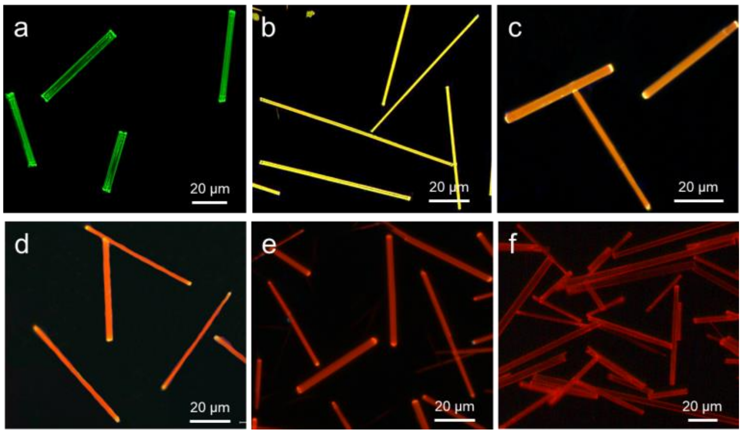

Supplementary Figure 8. FM images of BTP microwires with BTB doping ratio of (a) 0%, (b) 1%, (c) 2.5%, (d) 5%, (e) 7.5%, and (f) 100%. The scale bars are 20 μm.

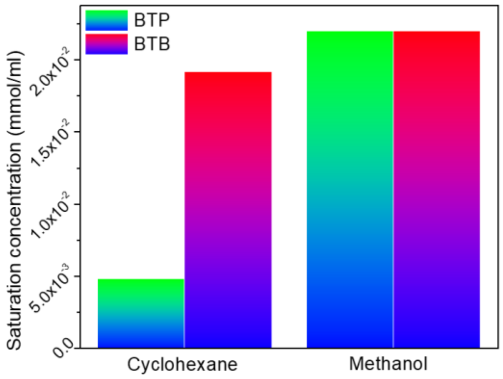

Supplementary Figure 9. The saturation concentration of BTP and BTB in cyclohexane and Methanol.

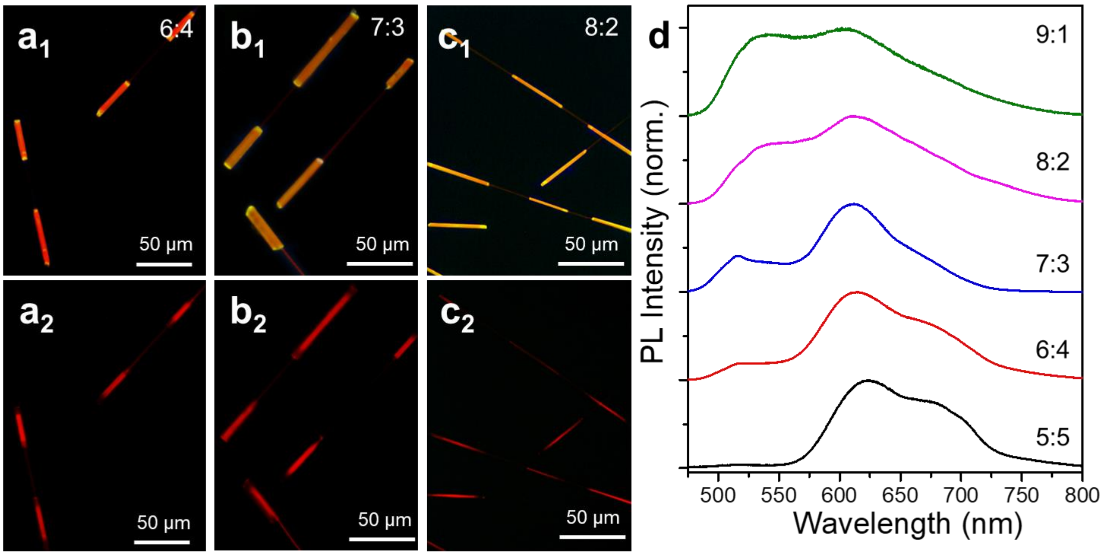

Supplementary Figure 10. FM images of the organic barcode microwires based on the different molar ratios between BTP and BTB cocrystal of (a) 6:4, (b) 7:3, and (c) 8:2. The scale bars are 50 μm. The microwires in (a_1, b_1, and c_1) and (a_2, b_2, and c_2) were respectively excited with UV light (λ = 330-380 nm) and green light (λ = 500-550 nm) from a mercury lamp. (d) Spatially resolved PL spectra of the organic barcode microwires based on the different molar ratios.

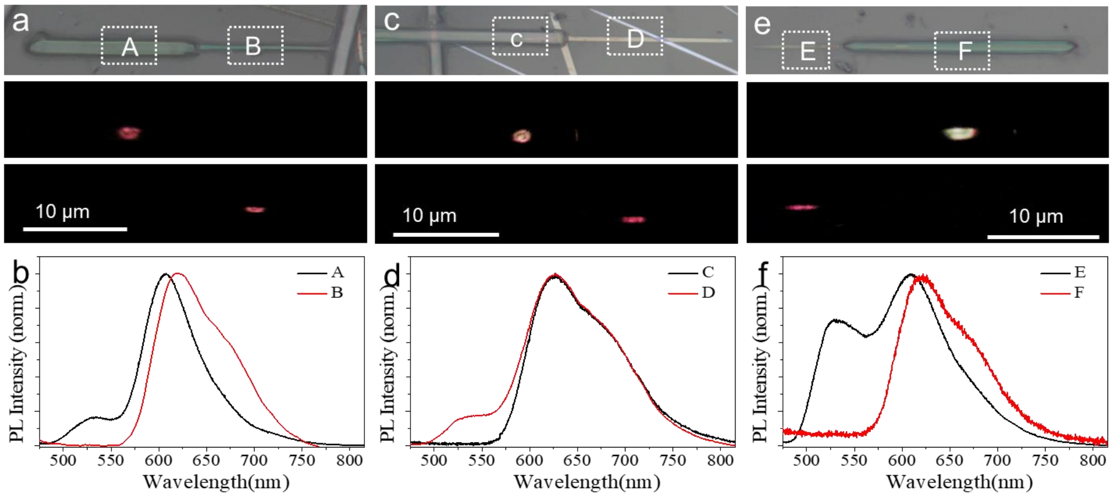

Supplementary Figure 11. (a) Bright-field of the typical organic barcode microwire, and corresponding FM image with A and B excited position. Scale bars are 10 μm. (b) Spatially resolved PL spectra of A and B excited position in the organic barcode microwires. (c) Bright-field of the typical organic barcode microwire, and corresponding FM image with C and D excited position. Scale bars are 5 μm. (d) Spatially resolved PL spectra of C and D excited position in the organic barcode microwires. (e) Bright-field of the typical organic barcode microwire, and corresponding FM image with E and F excited position. Scale bars are 10 μm. (f) Spatially resolved PL spectra of E and F excited position in the organic barcode microwires.

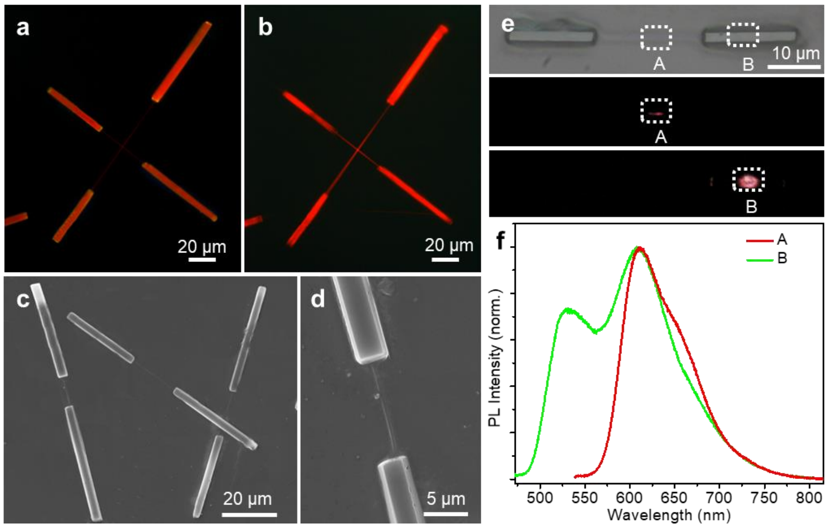

Supplementary Figure 12. (a and b) FM images of the typical organic barcoded heterostructures stored for 90 days with the excitation of (a) the UV band and (b) the green band (500–550 nm). The scale bars are 20 μm. (c and d) SEM images of the typical organic barcoded heterostructures stored for 90 days. The scale bars are 20 and 5 μm, respectively. (e) Bright-field and FM images of the individual organic barcoded heterostructure with a scale bar of 20 μm. (f) The Spatially resolved PL spectra collected from different locations marked in (e).

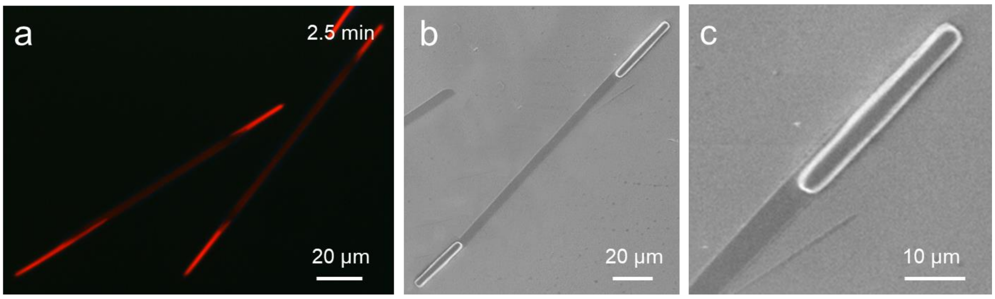

Supplementary Figure 13. (a) FM and (b and c) SEM images of the typical organic barcode microwires obtained at 2.5 min. The scale bars of (a and b) and (c) 20 and 10 μm, respectively.

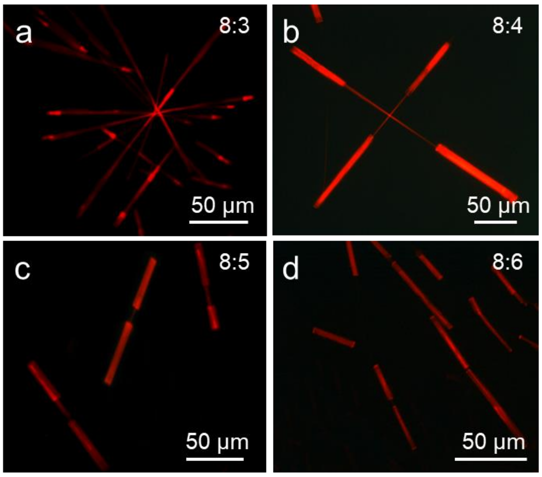

Supplementary Figure 14. FM images of the organic barcode microwires based on the different molar ratios between BTP and BTB cocrystal of (a) 8:3, (b) 8:4, (c) 8:5, and (d) 8:6 excited by green-light. The scale bars are 50 μm.

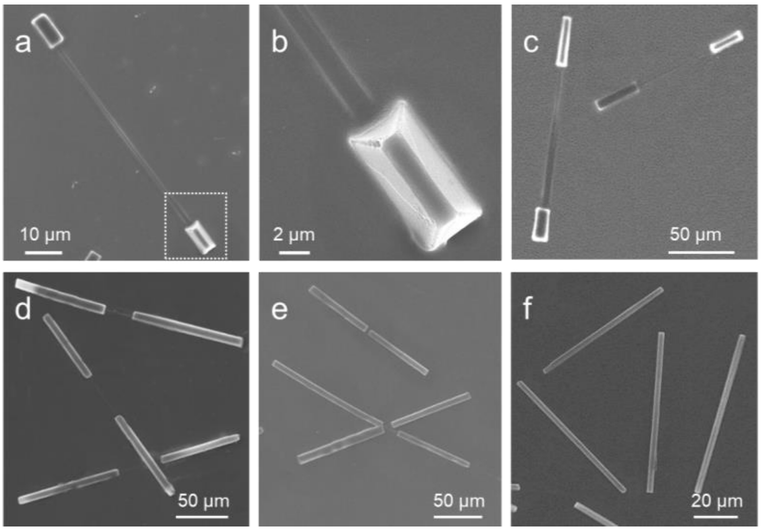

Supplementary Figure 15. SEM image of the individual organic barcode microwires based on the different molar ratios between BTP and BTB cocrystal of (a and b) 8:3, (c) 8:4, (d) 8:5, (e) 8:6, and (f) 8:8. The scale bar of (a), (b), (c-e), and (f) are 10, 2, 50 and 20 μm, respectively.

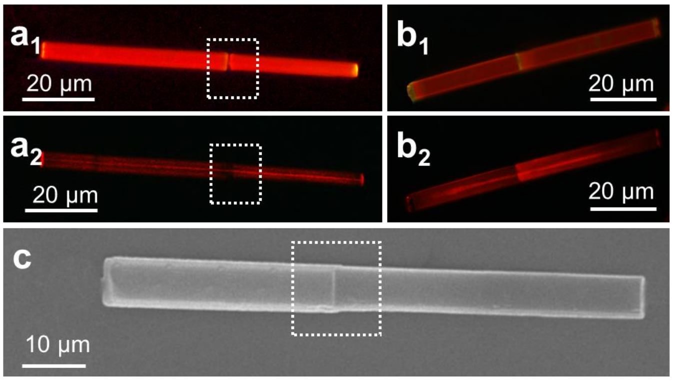

Supplementary Figure 16. (a and b) FM images of organic core-shell microwires excited by (a_1 and b_1) UV light and (a_2 and b_2) green light. The scale bars are 20 μm. (c) SEM images of organic core-shell microwires with scale bar of 10 μm.

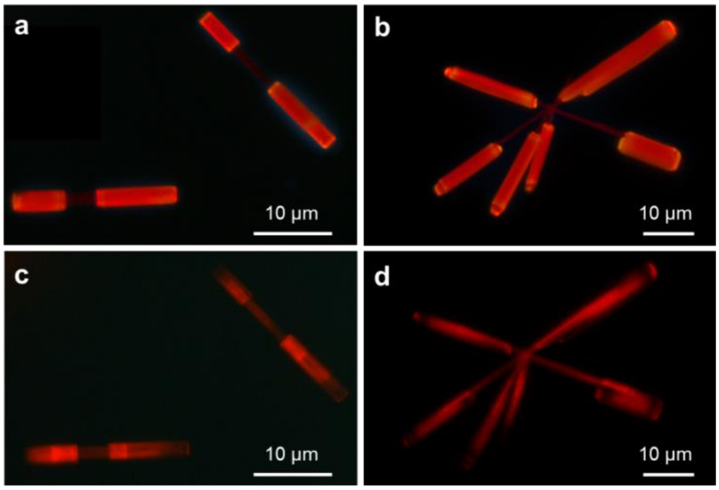

Supplementary Figure 17. FM images of the organic barcoded heterostructures stored prepared with concentration of (a and c) 5 mmol/L and (b and d) 15 mmol/L for the BTB and BTP stock solution in the hierarchical self-assembly. The corresponding excitation are (a and b) the UV band and (c and d) the green band (500-550 nm), respectively. The scale bars are 10 μm.

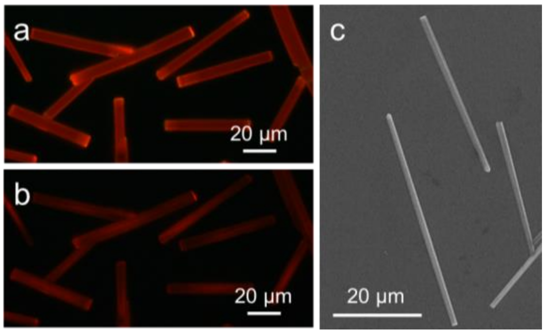

Supplementary Figure 18. (a and b) FM images of organic core-shell microwires prepared with a molar ratio η of 8:9 excited by (a) UV light and (b) green light. (c) The corresponding SEM image of these prepared organic core-shell microwires. All of these scale bars are 20 μm.

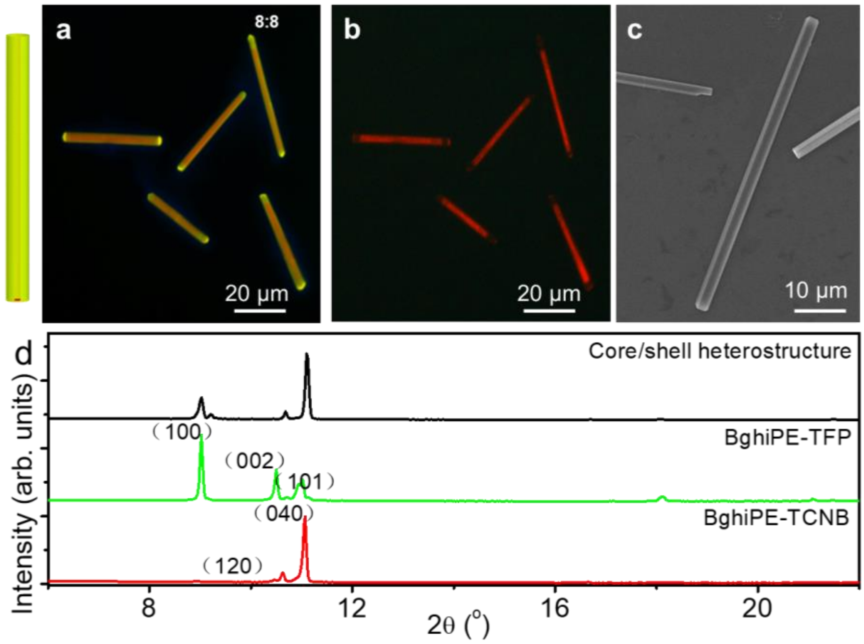

Supplementary Figure 19. (a and b) FM images of organic core-shell microwires excited by (a) UV light and (b) green light. The scale bars are 20 μm. (c) The corresponding SEM images of organic core-shell microwires with scale bar of 10 μm. (d) The XRD patterns of BTB microwires (red line), BTP microwires (green line), and core-shell heterostructure microwires (black line).

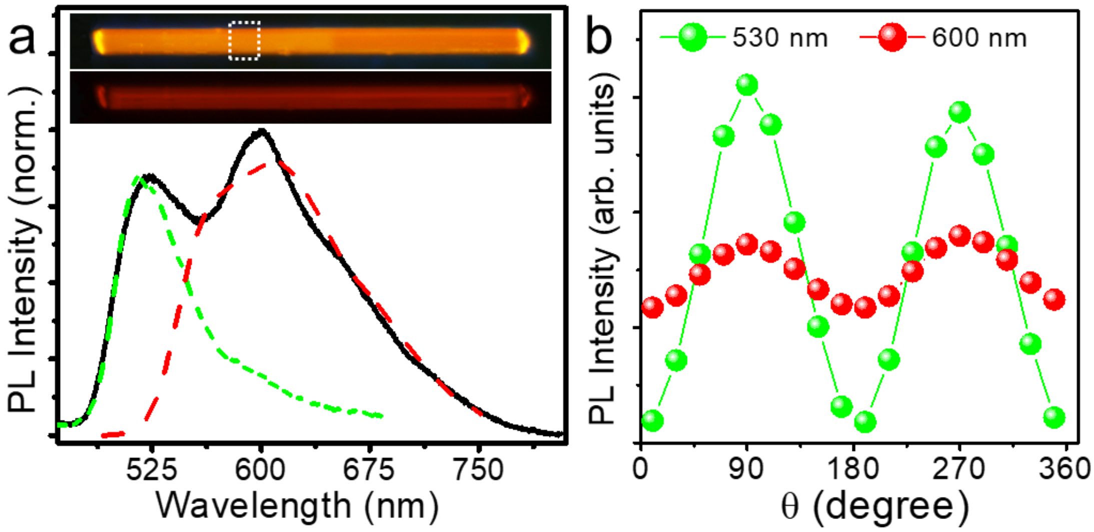

Supplementary Figure 20. (a) The spatially resolved PL spectra corresponding to different locations marked domains in the typical organic core-shell microwires in the inset. (b) The corresponding polar image of the PL peak intensities.

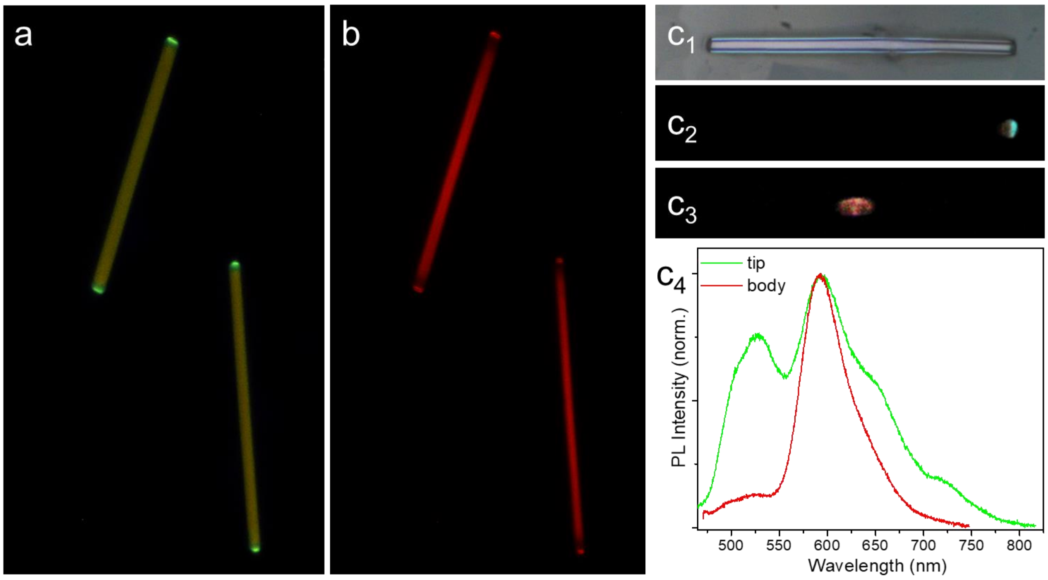

Supplementary Figure 21. (a and b) FM images of organic core-shell microwires prepared with a molar ratio η of 8:10. The excitation are (a) UV and (b) green light, respectively. The scale bars are 20 μm. (c_1) Bright-field and (c_2 and c_3) FM images of one typical organic triple-block microwire with (c_2) UV-light and (c_3) green-light excitation; and (c_4) the spatially resolved PL spectra corresponding to different locations marked domains in (c_1 and c_2).

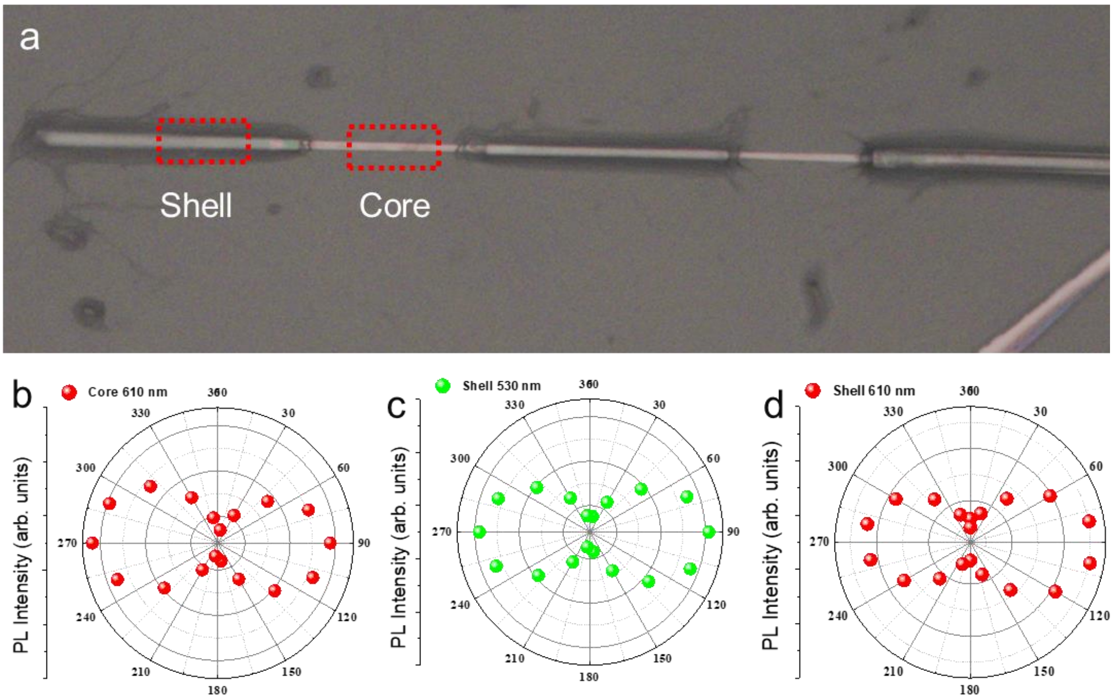

Supplementary Figure 22. (a) Bright-field image of organic barcode microwires. (c-d) The polar image of the PL peak intensities of organic barcode microwires in (a).

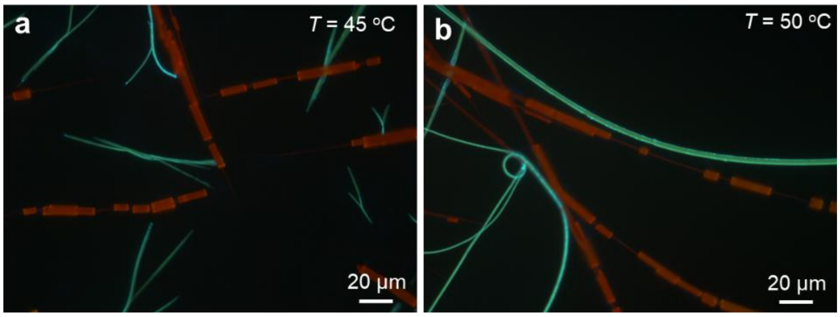

Supplementary Figure 23. (a and b) FM images of the organic microcrystals obtained via a horizontal epitaxial growth after increasing the temperature of the stock solution more than 40°C. The scale bars are 20 μm. The corresponding excitation is UV light.

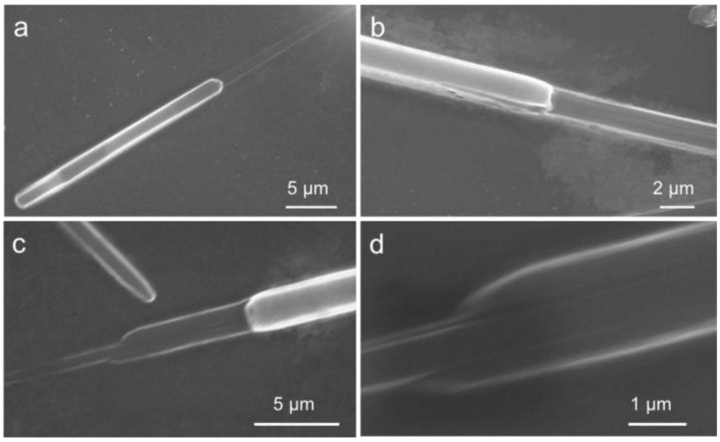

Supplementary Figure 24. SEM images of the typical tips in organic barcoded microwire. The scale bars of (a and c), (b), and (d) are 5, 2, and 1 μm, respectively.

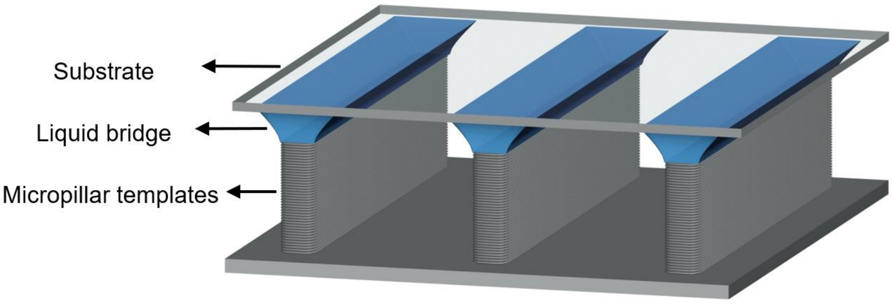

Supplementary Figure 25. Schematic illustration of the sandwich-type structure.

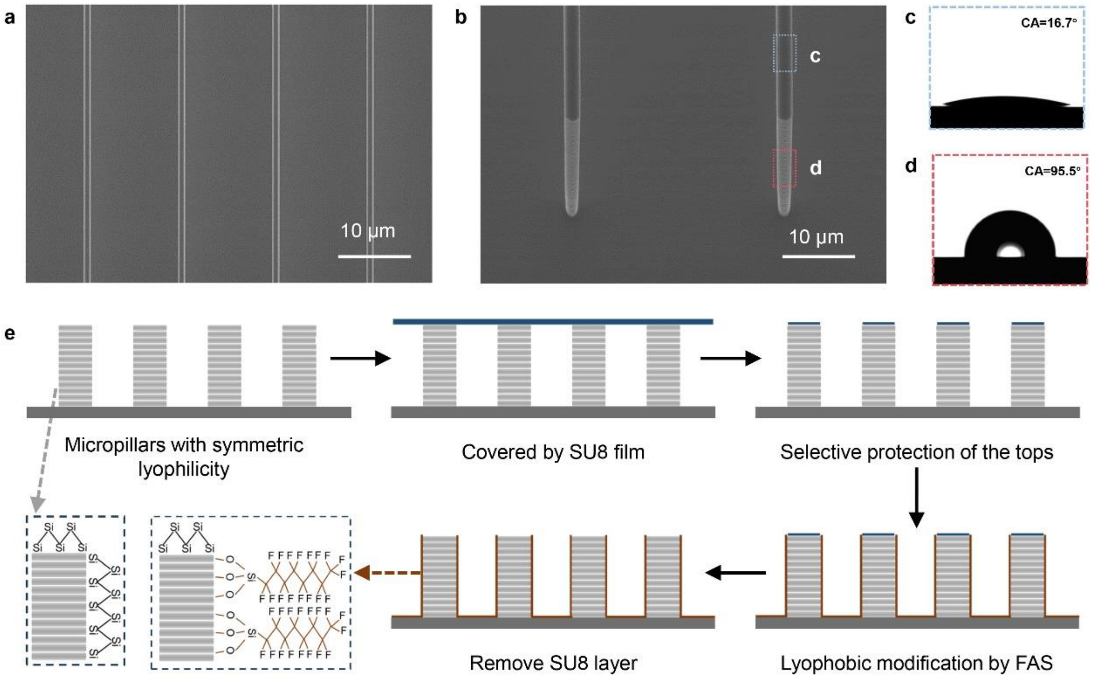

Supplementary Figure 26. Microwire array template with asymmetric wettability. (a) Top-view SEM image of the template with periodic line-shaped micropillars. (b) Side-view SEM image of the asymmetric-wettability template. (c and d) The contact angle (CA) for the top and sidewalls of micropillars. (e) Lyophobic modification of the pillar sidewalls. The scale bares are 10 µm.

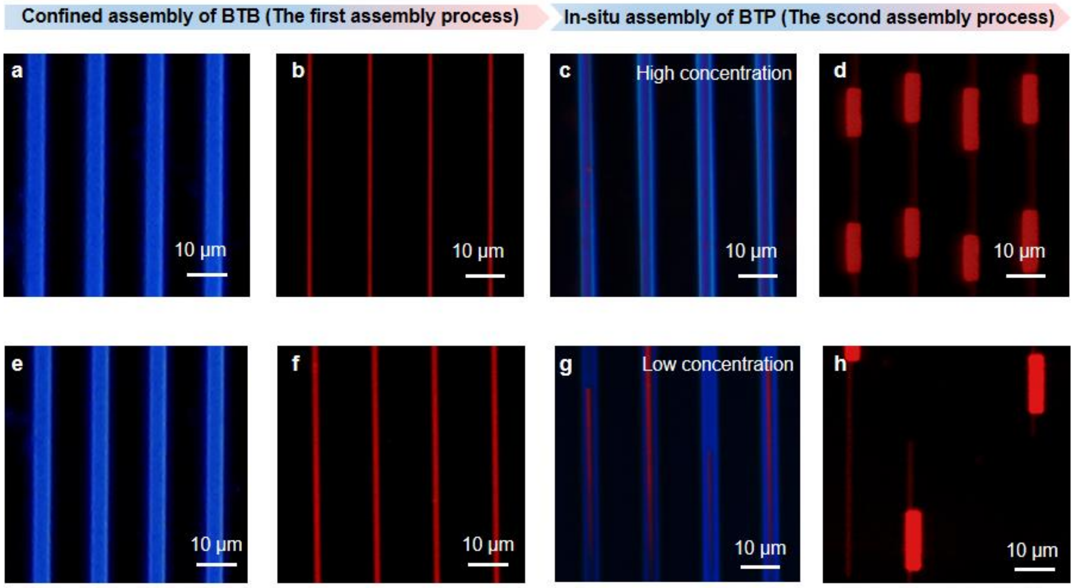

Supplementary Figure 27. FM images of the organic microstructures obtained in the capillary-bridge confined assembly method: (a and e) individual capillary bridges, (b and f) 1D BTB microwire arrays, (c and g) capillary bridges of BTP, (d and h) organic barcoded microwires. The concentration of BTP stock solution are (a-d) 20 mmol/L and (e-f) 10 mmol/L. The scale bares are 10 µm.

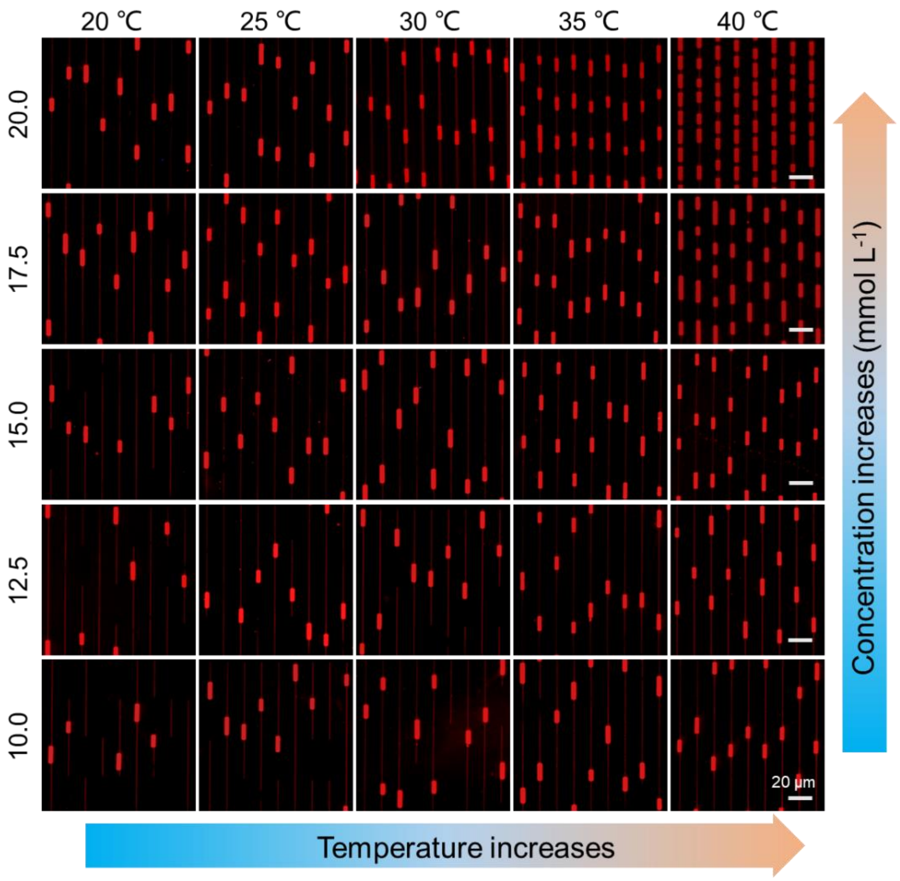

Supplementary Figure 28. FM images of the organic barcoded microwire arrays obtained in the capillary-bridge confined assembly method via finely adjusting the temperature and the concentration of BTP stock solution. The scale bars are 20 μm.

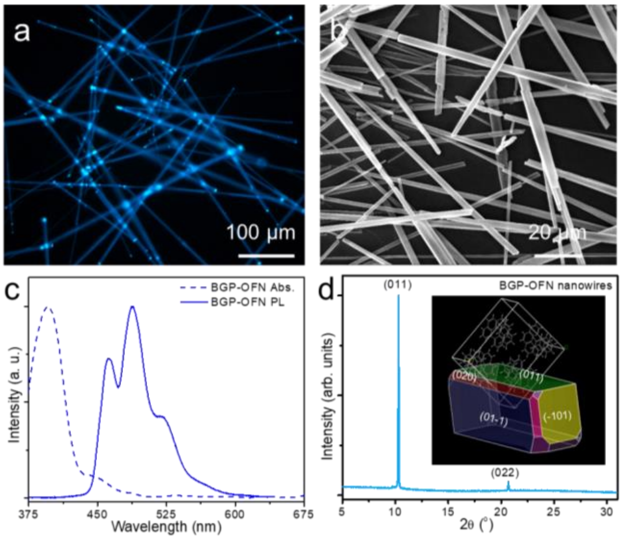

Supplementary Figure 29. (a) FM and (b) SEM images of BGP-OFN (BON) microwires. The scale bars of (a) and (b) are 100 and 20 μm, respectively. (c) The spatially resolved PL and absorption spectra of BON microwires. (d) The XRD pattern of BON microwires. Insert: predicted growth morphology.

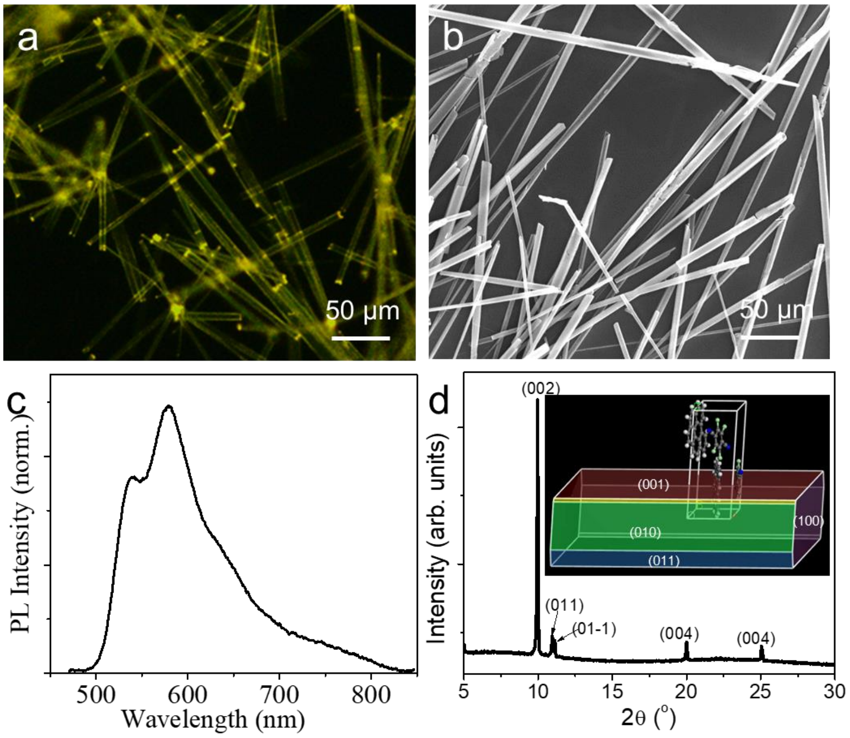

Supplementary Figure 30. (a) FM and (b) SEM images of BGP-TClP (BPP) microwires. The scale bars of (a) and (b) are 100 and 20 μm, respectively. (c) The spatially resolved PL and absorption spectra of BPP microwires. (d) The XRD pattern of BPP microwires. Insert: predicted growth morphology.

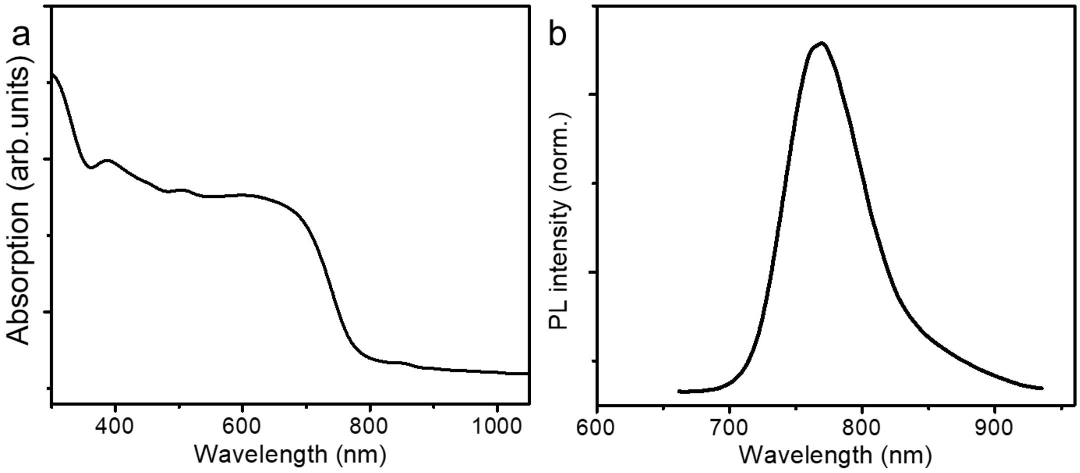

Supplementary Figure 31. (a) Absorption and (b) PL spectra of BcP-TCNQ cocrystal.

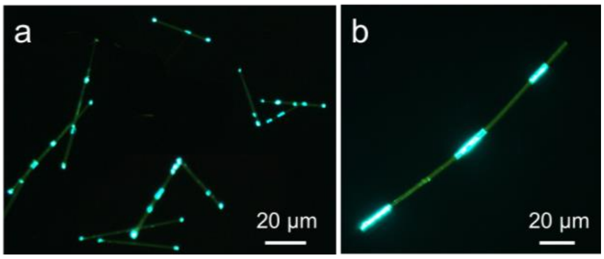

Supplementary Figure 32. FM images of blue-green based organic barcode microwires with the scale bars of 20 μm.

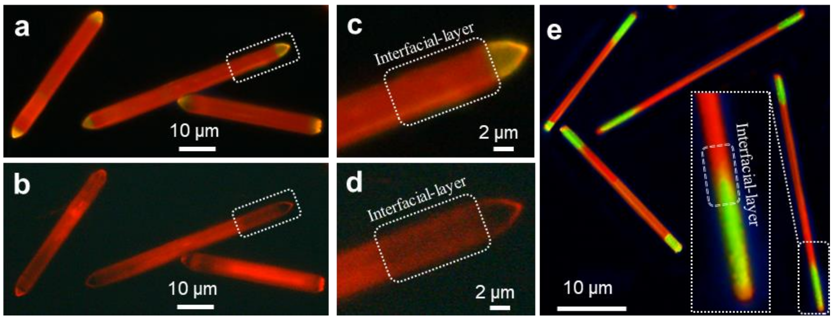

Supplementary Figure 33. FM images of the organic triblock microwires prepared via a sequential crystallization process (a-d) at the first state and (e) final state. The excitation are (a, c and e) the UV band and (b and d) the green band (500-550 nm). The scale bars are (a, b, and e) 10 μm and (c and d) 2 μm, respectively.

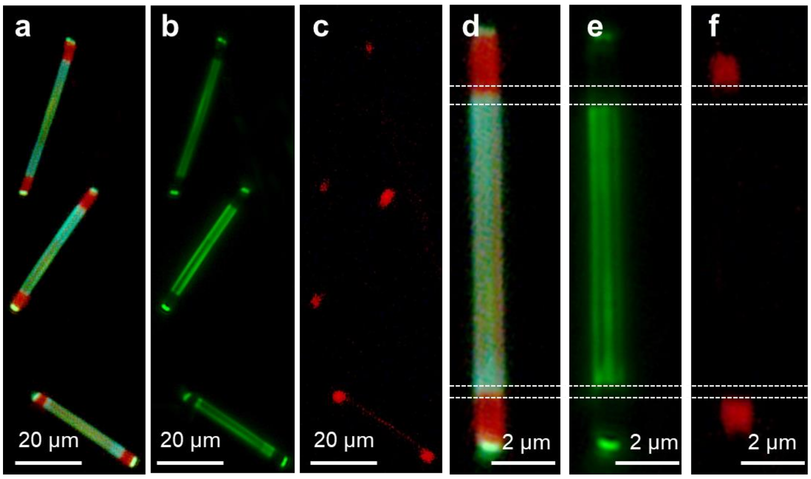

Supplementary Figure 34. FM images of organic barcoded microwires prepared by physical vapor deposition (PVD) method. Excitation are (a, and d) the UV band, (b, and e) blue light, and (c and f) green light, respectively. The scale bars are (a-c) 20 μm and (d-f) 5 μm.

Supplementary Table 1. Attachment energies and surface free energies of various crystal facets (hkl) of DMHP calculated by using the Materials Studio software package.

{hkl}	*d* (Å)		E_{attach} *(kcal/mol)*		$E_{surface}$ *(kcal/mol)*	
	BTB	BTP	BTB	BTP	BTB	BTP
{100}s	9.65	9.15		-36.20		18.16
{020}s	15.74	3.51	-34.29	-74.25	8.57	25.21
{001}s	7.13	16.45		-17.68		8.84
{110}s	9.22	5.57	-79.82	-66.42	40.02	34.85
{1-10}s	9.22	5.57	-79.82	-66.42	40.02	34.85
{011}s	6.96	6.46	-111.71	-55.79	56.41	28.30
{0-11}s	6.96	6.46	-111.71	-55.79	56.41	28.30
{101}s		8.00		-36.80		17.47
{10-1}s		8.00		-35.89		18.47

Supplementary References

1. Wang, Y. *et al.* Cocrystals Strategy towards Materials for Near-Infrared Photothermal Conversion and Imaging. *Angew. Chem. Int. Ed.* **57**, 3963-3967(2018).
2. Hu, W. *et al.* Cocrystal Engineering: toward Solution-Processed Near-Infrared 2D Organic Cocrystals for Broadband Photodetection. *Angew. Chem. Int. Ed.* **60**, 6344-6350 (2020).
3. Zhu, W. *et al.* Deepening Insights of Charge Transfer and Photophysics in a Novel Donor-Acceptor Cocrystal for Waveguide Couplers and Photonic Logic Computation. *Adv. Mater.* **28**, 5954-5962 (2016).
4. Zhao, Y. D. *et al.* Organic Charge-Transfer Cocrystals toward Large-Area Nanofiber Membrane for Photothermal Conversion and Imaging. *ACS Nano* **16**, 15000-15007 (2022).
5. Zhuo, M. P. *et al.* Segregated Array Tailoring Charge-Transfer Degree of Organic Cocrystal for the Efficient Near-Infrared Emission beyond 760 nm. *Adv. Mater.* **43**, 2107169 (2022).
6. Sun, Y., Lei, Y., Liao, L. & Hu, W. Competition between Arene-Perfluoroarene and Charge-Transfer Interactions in Organic Light-Harvesting Systems. *Angew. Chem. Int. Ed.* **56**, 10352-10356 (2017).
