# Supplementary material for: Visualizing the interfacial-layer-based epitaxial growth process toward organic core-shell architectures
Source: Nat Commun. 2024 Feb 7;15:1130. doi: 10.1038/s41467-024-45262-7 (PMC10850097; doi:10.1038/s41467-024-45262-7)
Supplement: Supplementary file 3 — Description of Additional Supplementary Files [file 41467_2024_45262_MOESM3_ESM.pdf]

Description of Additional Supplementary Files

File Name: Supplementary Data 1
Description: Chemical structure of 1,2,4,5-tetracyanobenzene.

File Name: Supplementary Data 2
Description: Chemical structure of benzo[ghi]perylene.

File Name: Supplementary Data 3
Description: Chemical structure of tetrafluoroterephthalonitrile.
